# Persisting Antibody Response to SARS-CoV-2 in a Local Austrian Population

**DOI:** 10.3389/fmed.2021.653630

**Published:** 2021-06-17

**Authors:** Dennis Ladage, Delia Rösgen, Clemens Schreiner, Dorothee Ladage, Christoph Adler, Oliver Harzer, Ralf J. Braun

**Affiliations:** ^1^Department of Internal Medicine, Danube Private University, Krems an der Donau, Austria; ^2^Heart Center, University of Cologne, Cologne, Germany; ^3^Department of Pneumology, Kliniken Maria Hilf, Moenchengladbach, Germany; ^4^Research Division for Neurodegenerative Diseases, Danube Private University, Krems an der Donau, Austria; ^5^Fire Department City of Cologne, Institute for Security Science and Rescue Technology, Cologne, Germany; ^6^Center of Biosciences, Danube Private University, Krems an der Donau, Austria; ^7^Bioscientia, Institute of Medical Diagnostics, Ingelheim am Rhein, Germany

**Keywords:** COVID-19, SARS-CoV-2, antibody, population, serotype

## Abstract

Severe acute respiratory syndrome coronavirus 2 (SARS-CoV-2) caused a global pandemic recently. The prevalence and persistence of antibodies following a peak SARS-CoV-2 infection provides insights into the potential for some level of population immunity. In June 2020, we succeeded in testing almost half of the population of an Austrian town with a higher incidence of COVID-19 infection. We performed a follow-up study to reassess the prevalence of SARS-CoV-2-specific IgA and IgG antibodies with 68 participants of the previous study. We found that the prevalence of IgG or IgA antibodies remained remarkably stable, with 84% of our cohort prevailing SARS-CoV-2-specific antibodies (only a slight decrease from 93% 4 months before). In most patients with confirmed COVID-19 seroconversion potentially provides immunity to reinfection. Our results suggest a stable antibody response observed for at least 6 months post-infection with implications for developing strategies for testing and protecting the population.

The world is still challenged by the severe acute respiratory syndrome coronavirus 2 (SARS-CoV-2) pandemic with the second wave culminating in autumn 2020 all over Europe, including Austria. It is still controversial, as to what extent and for how long previously affected people are immune to a recurring infection. During an infectious disease, B-lymphocytes produce immunoglobulin M (IgM) antibodies, which are later replaced by immunoglobulin A (IgA) and immunoglobulin G (IgG) antibodies. Persisting IgG antibodies are essential for developing a long-lasting immune response. In fact, more than 90% of people with known SARS-CoV-2 infections robustly develop antibodies to the SARS-CoV-2 spike protein, which comprises the receptor binding domain (RBD), enabling the virus to access human target cells (1–4). Thus, the antibody-based immune response is likely to play a decisive role in immunity toward SARS-CoV-2 infection.

In June 2020 (06/20/2020), we tested 835 participants, comprising 47% of the population of the Austrian town of Weißenkirchen in the Wachau, with a reported higher incidence of COVID-19 infection during the first wave in early spring 2020, and participants of less affected neighboring communities. In this pilot study (5), we used a sensitive enzyme-linked immunosorbent assay

(ELISA), enabling the semi-quantitative measurement of serum levels of IgG and IgA antibodies, specific for the RBD of the SARS-CoV-2 spike protein. We observed that 12% (98/835) of the tested were infected and consequently, developed SARS-CoV-2-specific IgG or IgA antibodies (5). Almost 9% (71/835) were positive for IgG antibodies and 9% (75/835) contained IgA antibodies. In June 2020, 6% (48/835) of our test population were serum-positive for both SARS-CoV-2-specific IgG and IgA antibodies (5).

In October 2020 (10/17/2020), we performed a follow-up study to reassess the prevalence of SARS-CoV-2-specific IgA and IgG antibodies in Weißenkirchen and neighboring communities. Blood samples were obtained to detect IgA and IgG antibodies specific for the RBD of the SARS-CoV-2 spike protein with a CE-certified laboratory-based ELISA method (Euroimmun Anti-SARS-CoV-2-ELISA IgG and IgA) performed in a certified diagnostic laboratory (Bioscientia, Ingelheim, Germany), as described in the pilot study (5). The study was conducted in accordance with the guidelines of the Local Ethics Committee and in approval of the local and national authorities. We specifically invited the 98 seropositive participants of the pilot study, but seronegative participants of the previous study were not excluded. In total we tested a group of 68 participants who had already participated in the pilot study.

Among the 68 participants, 93% (63/68) already tested positive in June 2020 (Figure 1A, left panel). Thus, our follow-up study comprised 64% (63/98) of the seropositive participants of the pilot study. In June 2020, 69% (47/68) of the patients were positive for IgG antibodies and 74% (50/68) contained IgA antibodies. Fifty percent (34/68) contained both IgG and IgA antibodies. In October 2020, we found in 84% (57/68) SARS-CoV-2-specific IgG or IgA antibodies (Figure 1B, right panel). Sixty-six percent (45/68) contained IgG antibodies and 74% (50/68) contained IgA antibodies. In 56% (38/68) of cases, both classes of antibodies were found. Thus, the prevalence of SARS-CoV-2-specific IgG and IgA antibodies remained extremely stable in the re-tested participants (Figure 1A, c.f. left and right panels). After four months, we found that 84% of our cohort still had SARS-CoV-2-specific antibodies, which is only a slight decrease from 93% in the previous test in June 2020.

**Figure 1:**
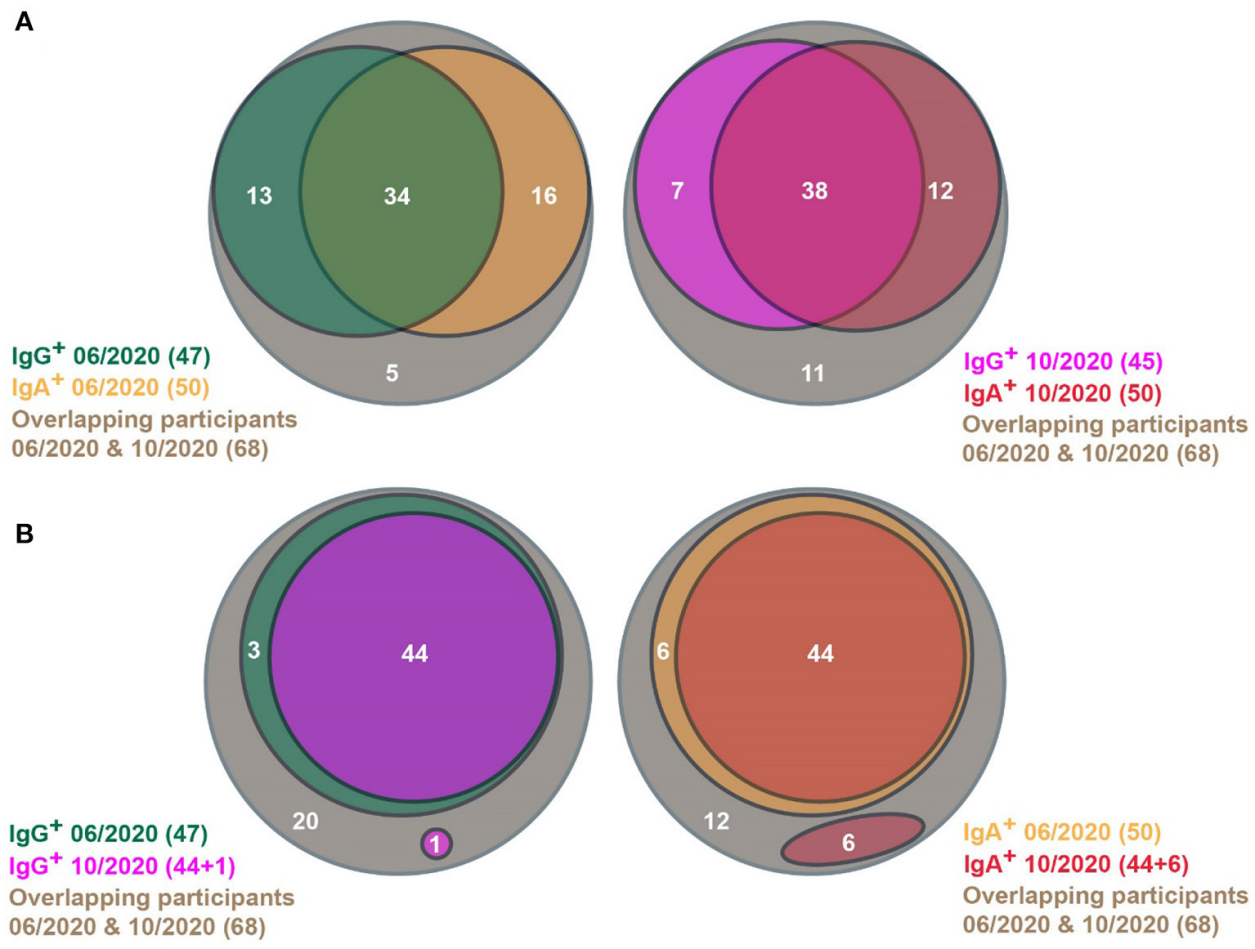
Venn diagrams showing SARS-CoV-2-specific antibody prevalence in the pilot (06/2020) and the follow-up (10/2020) studies. **(A)** SARS-CoV-2-specific antibody status of participants in the pilot (left) and the follow-up studies (right), respectively. **(B)** Persistence of SARS-CoV-2-specific IgG (left) and IgA antibodies (right), respectively, between the pilot and the follow-up studies. **(A,B)** Only people were considered, who participated in both studies.

This could be due to the high persistence of individual antibody responses. However, the antibody responses could wane in some individuals, which is superimposed by novel infections in other participants of the same subpopulation. Therefore, we analyzed the changes in antibody prevalence on an individual basis. Ninety-Four percentage (44/47) of people with SARS-CoV-2-specific IgG antibodies in June 2020 were still positive for IgG in October 2020 (Figure 1B, left panel). In one person, SARS-CoV-2-specific IgG antibodies could be found the first time in October 2020. Eighty-Eight percentage (44/50) of participants with SARS-CoV-2-specific IgA antibodies in June 2020 still contained marked IgA levels in October 2020 (Figure 1B, right panel). IgA antibody responses wer detected in October 2020 in six participants. Therefore, the continuance of antibody levels is only marginally influenced by novel infections.

When considering the alterations of antibody prevalence on an individual basis, the persistence of antibody responses remained very robust. Consequently, 97% (33/34) of participants with both SARS-CoV-2-specific IgG and IgA antibodies by June 2020, still contained significant levels of both classes of antibodies in October 2020 (Figure 2). Notably, the IgA antibody levels waned only in one of these participants, whereas the IgG antibody level remained significantly high in most. Only three persons with IgG (but lacking IgA) by June 2020 lost their IgG antibodies by October 2020. Surprisingly, five persons that lack IgA in June 2020 developed IgA by October 2020, then having both SARS-CoV-2-specific IgG and IgA antibodies. In five persons with IgA (but without IgG) in June 2020, their IgA antibodies waned by October 2020. Thus, the IgG antibody responses persisted very efficiently from June to October 2020, and the waning of the IgA antibody response was surprisingly low. One would expect a significant loss of the IgA antibodies because they are described as rather early and transient responders to an infection prior to the production of long-lasting IgG antibodies (6, 7). In contrast, in our study, a robust immune response with high levels of both SARS-CoV-2-specific IgG and IgA antibodies guaranteed the most efficient persistence of human antibody response, at least within the first 6 months after infection.

**Figure 2:**
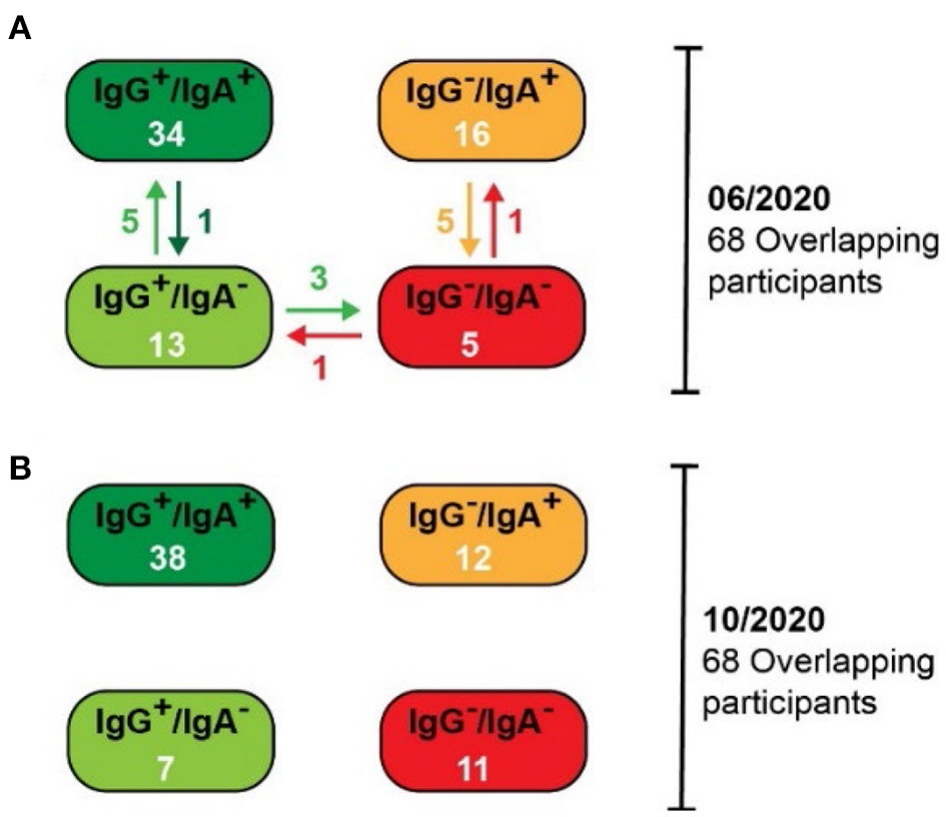
Alterations in the SARS-CoV-2-specific antibody prevalence between the pilot (06/2020) and the follow-up (10/2020) studies. **(A)** Antibody prevalence in the pilot study. Specific changes are indicated with arrows. **(B)** Antibody prevalence in the follow-up study.

The SARS-CoV-2-specific serum antibody levels may decrease over time in most individuals, but if the signals are above the threshold of the applied ELISA test system, this waning could be missed in our analysis so far. Therefore, we compared the relative IgG and IgA antibody levels from June 2020 to October 2020 for every participant (Figure 3). Using a semi-quantitative ELISA system, both IgG and IgA antibody levels hardly waned (on average 10% for IgG and 14% for IgA). Indeed, in some cases, we observed increased IgG and IgA antibody levels over time. Thus, these results support our notion that the antibody-based immune responses were very stable in the tested population between June and October 2020. Since most known COVID-19 cases in Weißenkirchen were noted in March 2020, our results suggest that the antibody-based immune responses last for more than 6 months. This may also have implications for the efficiency of SARS-CoV-2 vaccination. A strong antibody-based immune response involving both IgG and IgA antibodies upon vaccination may be predictive of immunity for more than 6 months after.

**Figure 3:**
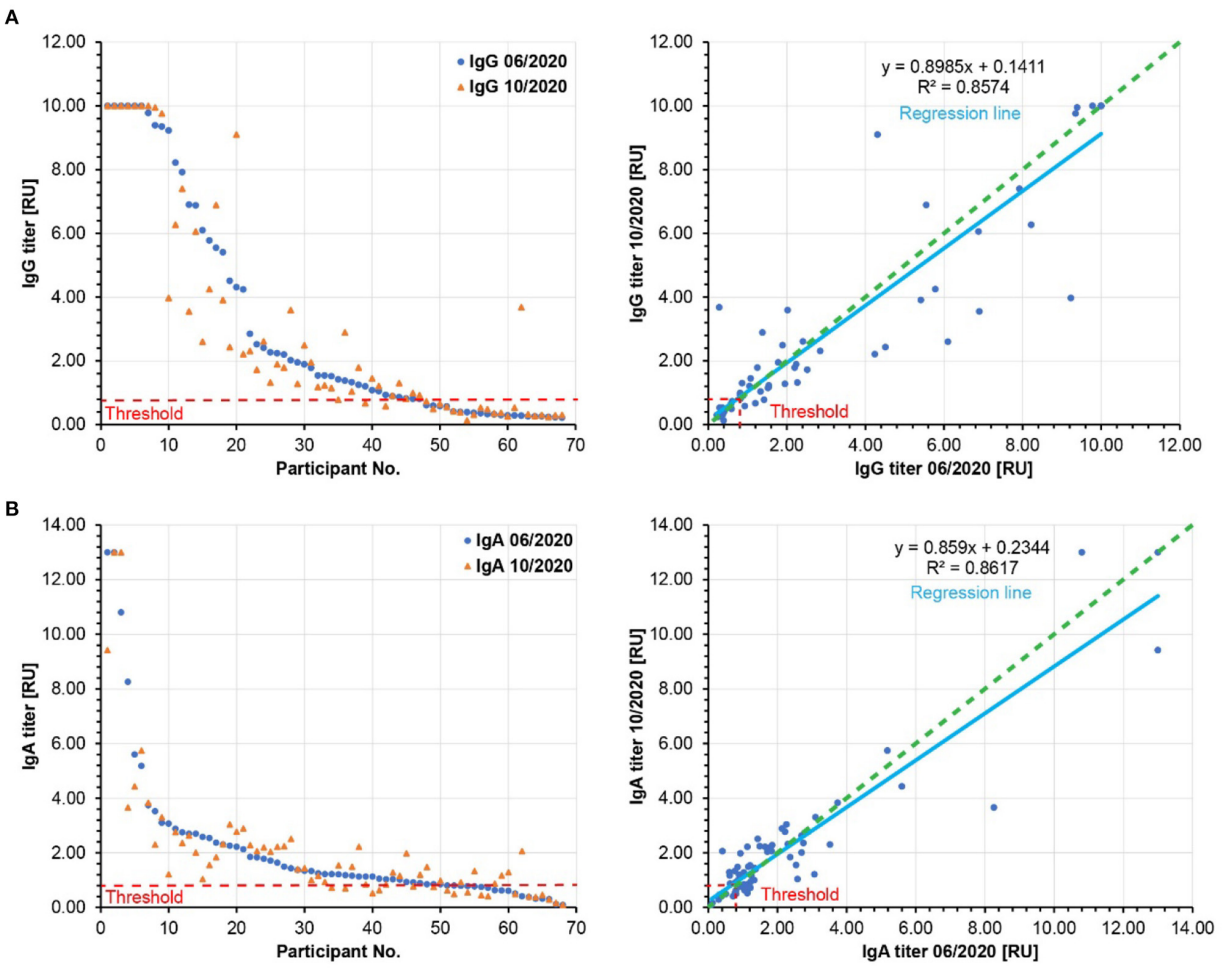
Relative SARS-CoV-2 specific IgG **(A)** and IgA **(B)** antibody titers. Left panels: The participants are ordered according to decreasing relative antibody titers of the pilot study (blue, 06/2020). The respective relative antibody titers of the follow-up study (10/2020) were plotted in orange. Right panels: The relative antibody titers of the follow-up study (10/2020) are plotted against the relative antibody titers of the pilot study (06/2020). The slopes of the regression lines (light blue) are below 1.0, showing a moderate waning to the relative antibody titers by 10% for IgG and by 14% for IgA. Green dashed lines: hypothetical regression lines in the case of 100% antibody persistence. Red dashed lines: Threshold of significant antibody detection (0.8 for both IgG and IgA). Only the data of the 68 participants are shown here, whose sera were analyzed in both pilot and follow-up studies.

The duration of SARS-CoV-2-specific antibodies persistence to provide immunity is still an open debate. Several studies suggest that the immune response persists for at least several months (6–12), whereas others propose rapid waning of the SARS-CoV-2-specific antibodies in the blood serum of previously infected individuals (2, 13). Although our study is limited by the small population size of our follow-up study, our findings support the idea of a prolonged immune response.

So far, studies determining antibody-based immune responses have been performed with either corona antibody rapid tests (which are less sensitive), or semi-quantitative ELISA tests (as in our study). Currently, ELISA methods for the quantitative assessment of SARS-CoV-2-specific IgG and IgA antibodies are emerging, allowing for a much more precise determination of antibody waning post-infection. In this study, samples were measured with both test systems in parallel for comparison of the semi-quantitative (see Figure 3) and quantitative analyses (data not shown and to be published later) in order to set a common base for subsequent studies.

In light of these technological advancements and the insufficient knowledge about the stability of SARS-CoV-2-triggered antibody-based immune responses, we will continue to test our cohort for SARS-CoV-2-specific IgG and IgA antibodies with both semi-quantitative and quantitative ELISA and combine these with novel tests for SARS-CoV-2-specific T-cell immunity. Waning of immune responses are expected, and we will test whether waning is influenced by age, sex, behavior (smoking, alcohol intake), weight, pre-existing conditions. We will also consider the role of the previous COVID-19 disease severity, as this has been proposed to influence the persistence of immunity with COVID-19 (14). To date, we have not detected any significant correlation between the persistence of antibody responses and these hallmarks. However, this may change when antibody waning becomes more relevant.

## Data Availability Statement

The raw data supporting the conclusions of this article will be made available by the authors, without undue reservation.

## Ethics Statement

The studies involving human participants were reviewed and approved by Kommission für wissenschaftliche Integrität und Ethik (Ethikkommission), Danube Private University, Krems an der Donau, Austria. The patients/participants provided their written informed consent to participate in this study.

## Author Contributions

DeL and RB analyzed the data and wrote the manuscript. OH, DR, CS, and DoL contributed to data analysis. OH and CA provided intellectual input. All authors contributed to the article and approved the submitted version.

## Conflict of Interest

The authors declare that the research was conducted in the absence of any commercial or financial relationships that could be construed as a potential conflict of interest.
